# Effect of bisphosphonate treatment of titanium surfaces on alkaline phosphatase activity in osteoblasts: a systematic review and meta-analysis

**DOI:** 10.1186/s12903-020-01089-4

**Published:** 2020-04-25

**Authors:** Christian Wehner, Stefan Lettner, Andreas Moritz, Oleh Andrukhov, Xiaohui Rausch-Fan

**Affiliations:** 1 Division of Conservative Dentistry and Periodontology, University Clinic of Dentistry, Medical University of Vienna, Sensengasse 2a, A-1090 Vienna, Austria; 2 Division of Oral Surgery, University Clinic of Dentistry, Medical University of Vienna, Vienna, Austria; 3 Austrian Cluster for Tissue Regeneration, Vienna, Austria

**Keywords:** Dental implants, Surface modification, Bisphosphonates, Osseointegration, Osteoblasts, Alkaline phosphatase activity

## Abstract

**Background:**

Bisphosphonate coating of dental implants is a promising tool for surface modification aiming to improve the osseointegration process and clinical outcome. The biological effects of bisphosphonates are thought to be mainly associated with osteoclasts inhibition, whereas their effects on osteoblast function are unclear. A potential of bisphosphonate coated surfaces to stimulate osteoblast differentiation was investigated by several in vitro studies with contradictory results. The purpose of this systematic review and meta-analysis was to evaluate the effect of bisphosphonate coated implant surfaces on alkaline phosphatase activity in osteoblasts*.*

**Methods:**

In vitro studies that assessed alkaline phosphatase activity in osteoblasts following cell culture on bisphosphonate coated titanium surfaces were searched in electronic databases PubMed/MEDLINE, Scopus and ISI Web of Science. Animal studies and clinical trials were excluded. The literature search was restricted to articles written in English and published up to August 2019. Publication bias was assessed by the construction of funnel plots.

**Results:**

Eleven studies met the inclusion criteria. Meta-analysis showed that coating of titanium surfaces with bisphosphonates increases alkaline phosphatase activity in osteoblasts after 3 days (*n* = 1), 7 (*n* = 7), 14 (*n* = 6) and 21 (*n* = 3) days. (7 days beta coefficient = 1.363, *p*-value = 0.001; 14 days beta coefficient = 1.325, p-value < 0.001; 21 days beta coefficient = 1.152, p-value = 0.159).

**Conclusions:**

The meta-analysis suggests that bisphosphonate coatings of titanium implant surfaces may have beneficial effects on osteogenic behaviour of osteoblasts grown on titanium surfaces in vitro. Further studies are required to assess to which extent bisphosphonates coating might improve osseointegration in clinical situations.

## Background

Nowadays, titanium dental implants demonstrate high long-term success rates and have become a standard treatment option for teeth replacement and prostheses support [1, 2]. An essential requirement for stable implant anchoring is the osseointegration process, which was first described by *Brånemark* et al. in the late 1960s and is defined as a direct functional and structural connection between the implant surface and living bone [3]. About one decade later, the concept of dental implant surface properties as a paramount element in osseointegration was introduced by Albrektsson [4]. Earlier research efforts were mainly focused on dental implant geometry intending to improve clinical outcome and long-term success. Later the focus of interest was shifted towards topographical and chemical modifications of implant surfaces. These modifications aimed to improve osseointegration through enhancement of the underlying biological processes [5, 6]. Surface characteristics like roughness or hydrophilicity affect proteins adsorption, cell adherence, proliferation, and differentiation, which are essential factors influencing the physiological processes during osseointegration [7, 8]. Titanium still is considered as a golden standard nowadays; however, alternative materials such as zirconia have raised interest due to almost similar osseointegration ability and hypothetically lower risk of peri-implantitis [9, 10]. Besides topographical characteristics and hydrophilicity, surface coating with drugs, proteins, growth factors or specific agents is now extensively investigated as a future tool in implantology [11, 12].

Bisphosphonates are antiresorptive drugs that influence bone metabolism mainly via inhibition of osteoclast recruitment, differentiation, and bone resorption activity [13]. Frequent indications of bisphosphonates include osteoporosis, Paget’s disease, skeletal metastases or osteogenesis imperfecta [14]. Members of the bisphosphonate family that are in common clinical use comprise alendronate, zoledronate, risedronate, ibandronate, and pamidronate [15]. After cellular uptake, bisphosphonates block the farnesyl pyrophosphate synthase, a key enzyme of the mevalonate pathway that is critical for osteoclast function [16]. Besides their inhibitory effect on osteoclasts and bone resorption, bisphosphonates may promote the processes of bone formation and enhance osteogenic differentiation of mesenchymal stem cells (MSCs) [17]. Bisphosphonates have been shown to support osseous wound healing and bone formation in the animal model [18, 19]. Dental implant bisphosphonate coatings are successfully applied as local drug delivery systems, demonstrating higher bone to implant contact (BIC) and peri-implant bone mineralization in the animal model [20, 21]. Bisphosphonate coated implants exhibit an increase in mechanical fixation in the human bone when compared to non-coated control [22]. Therefore, bisphosphonate coatings of titanium surfaces might also be beneficial for dental implant healing and osseointegration.

The formation of new bone around the dental implants is a complex process driven by osteoblasts and MSCs and precisely orchestrated by different cytokines and growth factors [23]. Alkaline phosphatase (ALP) is a widely used marker for early osteoblast differentiation in vitro and is crucial for bone formation [24, 25]. ALP increases the local concentration of inorganic phosphate and thus promotes mineralization processes [26]. Currently, literature investigating the impact of bisphosphonates on ALP in osteoblasts is contradictory, demonstrating either stimulating [27, 28] or inhibitory [29, 30] effects. The aim of this systematic review and meta-analysis was to assess the available in vitro evidence on the effect of bisphosphonate coated titanium surfaces on osteoblasts derived ALP activity. The significance of the effect of bisphosphonates coating on ALP activity was further tested by meta-analysis.

## Methods

This systematic review and meta-analysis were performed following the PRISMA statement (Preferred Reporting Items for Systematic Reviews and Meta-Analyses) [31] and Cochrane handbook [32]. A PICO (Population, Intervention, Comparison, Outcome) strategy was defined to evaluate scientific evidence. Studies were considered eligible under the following criteria: In vitro evaluation of titanium surfaces (excluding animal studies and clinical studies) (P) that were coated with bisphosphonates (excluding studies adding bisphosphonates as a substrate during cell culture) (I), compared to non-treated control (C), regarding ALP activity in osteoblasts that have been cultured on the surfaces (O).

### Search strategy

A systematic literature search without time restriction was performed by two independent researchers using three electronic databases: PubMed/MEDLINE, Scopus, and ISI web of science. The language was limited to English. The following medical subject headings (MeSH) terms and keywords were used for search strategies in MEDLINE via PubMed: ((((((((bisphosphonate [MeSH Terms] OR bisphosphonate coating) OR phosphonate) OR alendronate) OR zoledronate) OR zoledronic acid) OR risedronate) OR ibandronate) OR pamidronate) AND (titanium OR titanium surface) AND ((alkaline phosphatase [MeSH Terms] OR alkaline phosphatase activity) OR ALP) AND (osteoblast [MeSH Terms] OR osteoblast-like cell). For ISI Web of Science and Scopus, the following search terms were used: (“bisphosphonate” OR “bisphosphonate coating” OR “alendronate” OR “zoledronate” OR “zoledronic acid” OR “risedronate” OR “ibandronate” OR “pamidronate”) AND (“titanium” OR “titanium surface”) AND (“alkaline phosphatase” OR “alkaline phosphatase activity” OR “ALP”) AND (“osteoblast” OR “osteoblast-like cell”).

### Inclusion criteria

Studies were included if they met the following criteria:

1. In vitro studies evaluating ALP activity in osteoblasts growing on titanium surfaces that were coated by bisphosphonates.
2. Studies written in English were included up until August 2019.
3. Sufficient data provided to perform calculations for the meta-analysis. In case data were not presented in the paper, the corresponding author was asked via e-mail to provide missing data. If there was no reply, measurement of the graphs by available online tools (GetData Graph Digitizer) that have been recommended by the Cochrane Handbook for Systematic Reviews of Interventions [33] was performed.

### Data extraction

Data extraction was carried out independently by two researchers (CW and OA). Each study was first checked regarding title, followed by screening of the abstracts and the full text. If the inclusion criteria were met, the following data were extracted for conduction of the meta-analysis: First author’s name, year of publication, sample size per experiment, time of ALP activity measurement, cell type used for experiments, measure of variability, type of bisphosphonate used for coating, amount or concentration of bisphosphonate on titanium surface, alkaline phosphatase activity, coating specification. To ensure data quality, studies were checked for description of methodology and a clearly focused research question. Furthermore, the presence of the following parameters was reviewed in each study to perform quality assessment: stability of bisphosphonate coating, quality of ALP activity assessment, description of coating procedure, availability of original data, surface roughness parameters, contact angle measurement, appropriate statistical analysis, and performance of at least three repetitions. If the required information was stated within the paper, the study received one point on that specific parameter. Study quality was assessed according to the sum of points achieved: 1–3 = high, 4–5 = medium, 6–8 = low quality. Any disagreements regarding study eligibility were discussed and solved by consulting a third researcher (XR).

### Statistical analysis

For the meta-analysis, synthesis of the studies was carried out using the response ratio [34], which was calculated as the ratio of ALP activity value measured in the treatment group to those measured in the control group. This was done to avoid the effect of the variability of the absolute ALP activity values between the studies, which might depend on the used protocol and cell type. Calculations were done using the log of this ratio, but for the presentation, the results were back-transformed using the exponential function. Random-effects models were used to account for the high heterogeneity in the included studies. Additionally, multilevel models were necessary to account for including several groups of the same study in the analysis. Thus, meta-analytic multilevel random-effects models [35] were used, including a random effect for the studies. Tests and confidence intervals from these models presented are based on Wald statistics.

### Risk of publication bias

Funnel plots on the log response ratio scale, as well as forest plots, were prepared. Publication bias was assessed by visually inspecting funnel plots and calculating Egger’s test egger [36] and Kendall’s tau [37] according to the suggestion of The PRISMA Statement for Reporting Systematic Reviews and Meta-Analyses [38]. Heterogenity was quantified using I^2^ as defined by Higgins & Thompson 2002 [39]. All computations were done using R version 3.5.1.(R: A language and environment for statistical computing).

No further risk of bias was assessed as no validated bias risk assessment tool was available for in vitro studies.

## Results

### Screening process and study selection

**Fig. 1:**
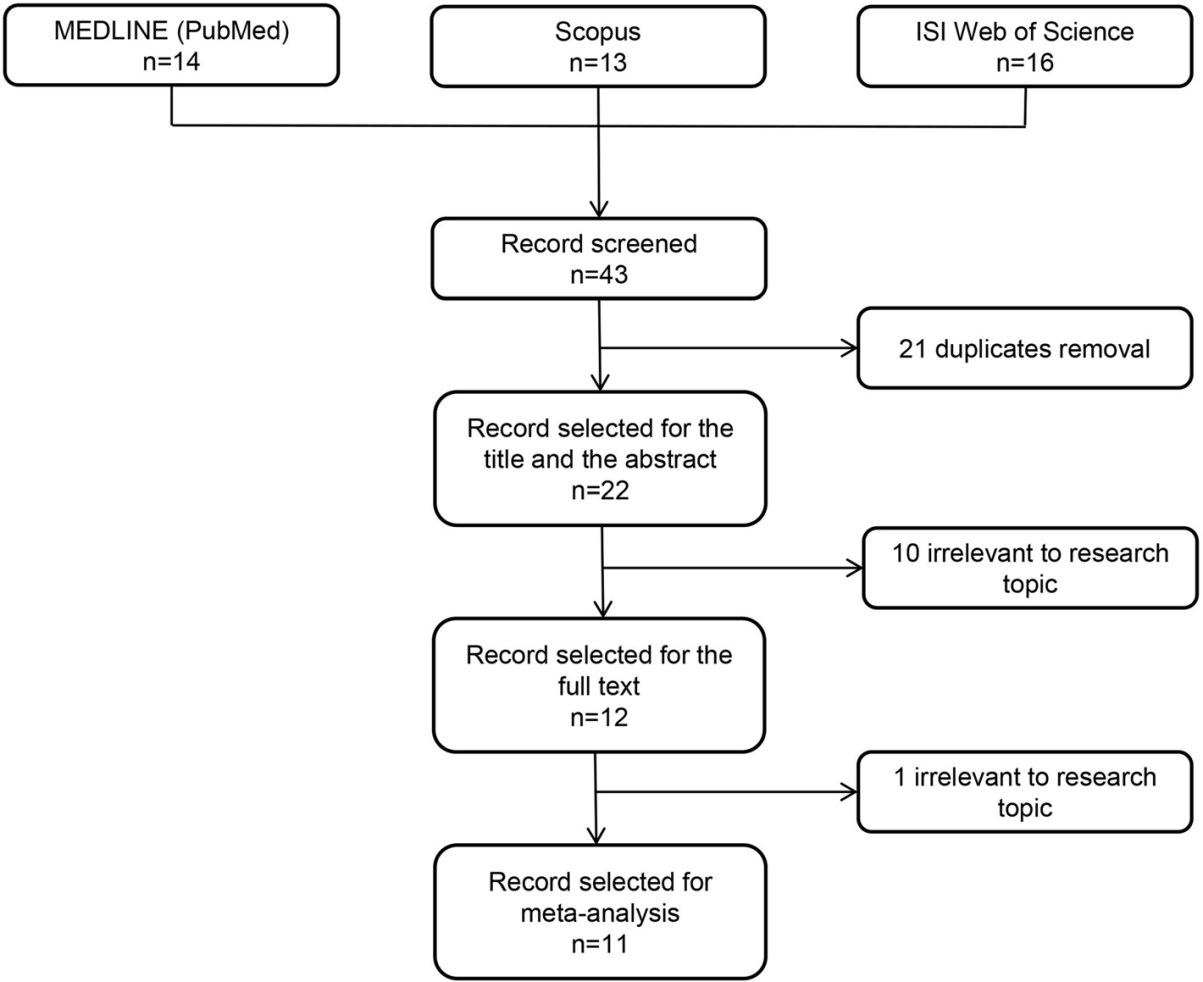
Flowchart of the study screening process according to PRISMA statement

The flowchart of the screening process is presented in Fig.1. The literature reviewing process revealed 42 studies: 14 from MEDLINE (PubMed), 16 from ISI Web of Science, 13 from Scopus electronic database. Twenty-two studies remained after duplicates removal. Of those, 11 studies had to be excluded as they did not match the following PICO criteria: P (*n* = 2), I (*n* = 6), O (*n* = 3). Finally, 11 studies were enrolled for meta-analytic calculations.

### Descriptive analysis

Table 1 shows the characteristics of the included studies for meta-analysis. All 11 studies were published between 2000 and 2019. In five studies experiments were performed with MG-63 human osteoblast-like cells [40, 41, 43, 45, 46], in three studies with MC3T3-E1 mouse osteoblast cells [44, 47, 50], in two studies with osteoblasts from rat calvaria [48, 49], and in one study with osteoblasts derived from primary human stem cells [42]. The sample size per experiment was ranging between 3 and 6. ALP activity was measured after 3, 4, 7, 10, 14, 18 or 21 days of cell culture; most studies (*n* = 7) performing experiments after 1 week. Nine studies used alendronate as coating, one study zoledronate, and one pamidronate.

**Table 1:** Study characteristics of the included studies. ALP activity: alkaline phosphatase activity, SrHA: *strontium* hydroxyapatite, and ZOLHA (*zoledronate* hydroxyapatite), BMP-2: *bone morphogenetic protein 2*

Study ID	Year	Sample size per experiment	Time point of measurement	Cell type	Type of bisphosphonate	Amount / concentration	ALP activity (3 days)	ALP activity (4 days)	ALP activity (7 days)	ALP activity (10 days)	ALP activity (14 days)	ALP activity (18 days)	ALP activity (21 days)	Coating specificaiton
**Bigi et al.** [40]	2009	3	7, 14 days	MG-63 osteoblast like cells	Control	–			2.17 ± 1.21		4.81 ± 0.27			Hydroxyapatite
**Bigi et al.** [40]	2009	3	7, 14 days	MG-63 osteoblast like cells	Alendronate	7 mM			2.12 ± 0.20		4.72 ± 0.08			Hydroxyapatite
**Bigi et al.** [40]	2009	3	7, 14 days	MG-63 osteoblast like cells	Alendronate	28 mM			2.29 ± 0.45		5.23 ± 0.14			Hydroxyapatite
**Boanini et al.** [41]	2015	6	10, 21 days	MG-63 osteoblast like cells	Control	–				0.82 ± 0.12			0.99 ± 0.12	Strontium-substituted hydroxyapatite (SrHA)
**Boanini et al.** [41]	2015	6	10, 21 days	MG-63 osteoblast like cells	Zoledronat	Gradient composition C2 (SrHA and ZOLHA)				1 ± 0.15			1.09 ± 0.08	Strontium-substituted hydroxyapatite (SrHA)
**Boanini et al.** [41]	2015	6	10, 21 days	MG-63 osteoblast like cells	Zoledronat	Gradient composition C3 (SrHA and ZOLHA)				0.79 ± 0.1			0.92 ± 0.08	Strontium-substituted hydroxyapatite (SrHA)
**Boanini et al.** [41]	2015	6	10, 21 days	MG-63 osteoblast like cells	Zoledronat	Gradient composition C4 (SrHA and ZOLHA)				0.75 ± 0,1			0.86 ± 0.16	Strontium-substituted hydroxyapatite (SrHA)
**Boanini et al.** [41]	2015	6	10, 21 days	MG-63 osteoblast like cells	Zoledronat	ZOLHA				0,80 ± 0.06			0.92 ± 0,15	Strontium-substituted hydroxyapatite (SrHA)
**Boanini et al.** [42]	2015	6	14 days	Osteoblast derived from stem cells	Control	–					10.6 ± 2.1			Octacalcium phosphate
**Boanini et al.** [42]	2015	6	14 days	Osteoblast derived from stem cells	Alendronate	8 mM					16.3 ± 2.6			Octacalcium phosphate
**Boanini et al.** [42]	2015	6	14 days	Osteoblast derived from stem cells	Alendronate	20 mM					9.63 ± 2.30			Octacalcium phosphate
**Hu et al.** [44]	2013	5	14 days	MC3T3-E1	Control	–					0.41 ± 0.02			Precoated hydroxyapatite (CaP) layer
**Hu et al.** [44]	2013	5	14 days	MC3T3-E1	Alendronate	0.2 mg/ml solution					0.57 ± 0.04			Precoated hydroxyapatite (CaP) layer
**Hu et al.** [44]	2013	5	14 days	MC3T3-E1	Alendronate	0.5 mg/ml solution					0.59 ± 0.04			Precoated hydroxyapatite (CaP) layer
**Hu et al.** [44]	2013	5	14 days	MC3T3-E1	Alendronate	1 mg/ml solution					0.59 ± 0.63			Precoated hydroxyapatite (CaP) layer
**Jeon et al** [43]	2019	5	3 days	MG-63 osteoblast like cells	Control	–	1.11 ± 0.09							UV treatment
**Jeon et al** [43]	2019	5	3 days	MG-63 osteoblast like cells	Alendronate	10^−3^ M	1.16 ± 0.17							UV treatment
**Kim et al**. [45]	2013	5	7, 14, 21 days	MG-63 osteoblast like cells	Control	–			1.12 ± 0.03		3.44 ± 0.14		4.01 ± 0.09	Heparin-coated
**Kim et al**. [45]	2013	5	7, 14, 21 days	MG-63 osteoblast like cells	Alendronate	1 mg/ml solution			1.19 ± 0.04		5.29 ± 0.06		4.41 ± 0.12	Heparin-coated
**Kim et al**. [45]	2013	5	7, 14, 21 days	MG-63 osteoblast like cells	Control	–			1.31 ± 0.05		5.86 ± 0.23		4.65 ± 0.13	BMP-2/Heparin-coated
**Kim et al**. [45]	2013	5	7, 14, 21 days	MG-63 osteoblast like cells	Alendronate	1 mg/ml solution			1.41 ± 0.04		6.65 ± 0.27		5.36 ± 0.13	BMP-2/Heparin-coated
**Kim et al.** [46]	2017	3	7 days	MG-63 osteoblast like cells	Control	–			1.49 ± 0.23					without UV treatment
**Kim et al.** [46]	2017	3	7 days	MG-63 osteoblast like cells	Alendronate	10^−6^ M			4.08 ± 0.23					without UV treatment
**Kim et al.** [46]	2017	3	7 days	MG-63 osteoblast like cells	Control	–			3.49 ± 0.34					with UV treatment
**Kim et al.** [46]	2017	3	7 days	MG-63 osteoblast like cells	Alendronate	10^−6^ M			6.22 ± 0.78					with UV treatment
**Moon et al.** [47]	2011	3	7, 14, 21 days	MC3T3-E1	Control	–			2.56 ± 0.17		3.60 ± 0.06		3.48 ± 0.13	Heparin-coated
**Moon et al.** [47]	2011	3	7, 14, 21 days	MC3T3-E1	Alendronate	1 mg			3.55 ± 0.10		5.35 ± 0.17		4.36 ± 0.11	Heparin-coated
**Moon et al.** [47]	2011	3	7, 14, 21 days	MC3T3-E1	Alendronate	5 mg			4.24 ± 0.29		7.80 ± 0.21		5.35 ± 0.15	Heparin-coated
**Mu et al.** [48]	2018	5	7 days	Osteoblasts from neonate rat calvaria	Control	–			0.53 ± 0.05					Hyaluronan
**Mu et al.** [48]	2018	5	7 days	Osteoblasts from neonate rat calvaria	Alendronate	500 mg			0.63 ± 0.06					Hyaluronan
**Yoshinari et al.** [49]	2000	6	7 days	Osteoblastic cells from calvariae of Sprague-Dawley rats	Control	–			51.7 ± 5.9					Hydroxyapatite
**Yoshinari et al.** [49]	2000	6	7 days	Osteoblastic cells from calvariae of Sprague-Dawley rats	Pamidronate disodium	10^−2^ M			65.4 ± 9.7					Hydroxyapatite
**Zheng et al.** [50]	2016	3	4,7,10,14,18 days	MC3T3-E1	Control	–		1.19 ± 0.15	1.81 ± 0.20	3.27 ± 0,38	3.30 ± 0.27	2.66 ± 0.25		Plasma treated titanium
**Zheng et al.** [50]	2016	3	4,7,10,14,18 days	MC3T3-E1	Alendronate	2.5 mg/ml solution		1.59 ± 0.13	2.36 ± 0.27	3.58 ± 0.20	3.66 ± 0.28	2.65 ± 0.25		Plasma treated titanium
**Zheng et al.** [50]	2016	3	4,7,10,14,18 days	MC3T3-E1	Control	–		1.20 ± 0.16	1.95 ± 0,26	3.26 ± 0.16	3,3 ± 0.13	2.62 ± 0.36		Plasma treated, silane-treated
**Zheng et al.** [50]	2016	3	4,7,10,14,18 days	MC3T3-E1	Alendronate	0.5 mg/ml solution		1.52 ± 0.16	2.66 ± 0,16	3.93 ± 0.26	4.32 ± 0.30	3.18 ± 0.24		Plasma treated, silane-treated
**Zheng et al.** [50]	2016	3	4,7,10,14,18 days	MC3T3-E1	Alendronate	1 mg/ml solution		1.62 ± 0.12	3.39 ± 0,23	4.99 ± 0.28	3.70 ± 0.13	3.05 ± 0.14		Plasma treated, silane-treated

Study quality assessment is presented in Table 2. According to the criteria applied, 8 studies were classified as medium quality, and 3 studies with low quality, respectively. Quantitative data required for the analysis were provided in one out of 11 studies [46], four authors provided data upon e-mail request [40–42, 48]. In the remaining six studies [44, 45, 47, 49, 50], measurement of the graphs by available online software was performed because there was no response after e-mail request.

**Table 2:** Study quality assessment. 1) stability of bisphosphonate coating, 2) ALP measurement quality, 3) description of coating procedure, 4) availability of original data, 5) surface roughness parameters, 6) contact angle measurement, 7) appropriate statistical analysis, 8) performance of at least three repetitions per experiment

	1	2	3	4	5	6	7	8	Study quality
**Bigi et al.** [40]	No	No	Yes	Yes	Yes	No	Yes	Yes	Medium
**Boanini et al.** [41]	No	No	Yes	Yes	Yes	No	Yes	No	Medium
**Boanini et al.** [42]	Yes	No	Yes	Yes	Yes	No	Yes	No	Medium
**Hu et al.** [44]	Yes	Yes	Yes	No	No	Yes	Yes	Yes	Low
**Jeon et al.** [43]	No	Yes	Yes	No	No	No	Yes	Yes	Medium
**Kim et al**. [45]	Yes	Yes	Yes	No	No	No	Yes	Yes	Medium
**Kim et al.** [46]	No	Yes	Yes	Yes	No	No	Yes	No	Medium
**Moon et al.** [47]	Yes	Yes	Yes	No	No	Yes	Yes	Yes	Low
**Mu et al.** [48]	Yes	Yes	Yes	Yes	Yes	No	No	No	Medium
**Yoshinari et al.** [49]	No	Yes	Yes	No	Yes	No	Yes	No	Medium
**Zheng et al.** [50]	Yes	Yes	Yes	No	Yes	Yes	Yes	Yes	Low

### Meta-analysis

**Fig. 2:**
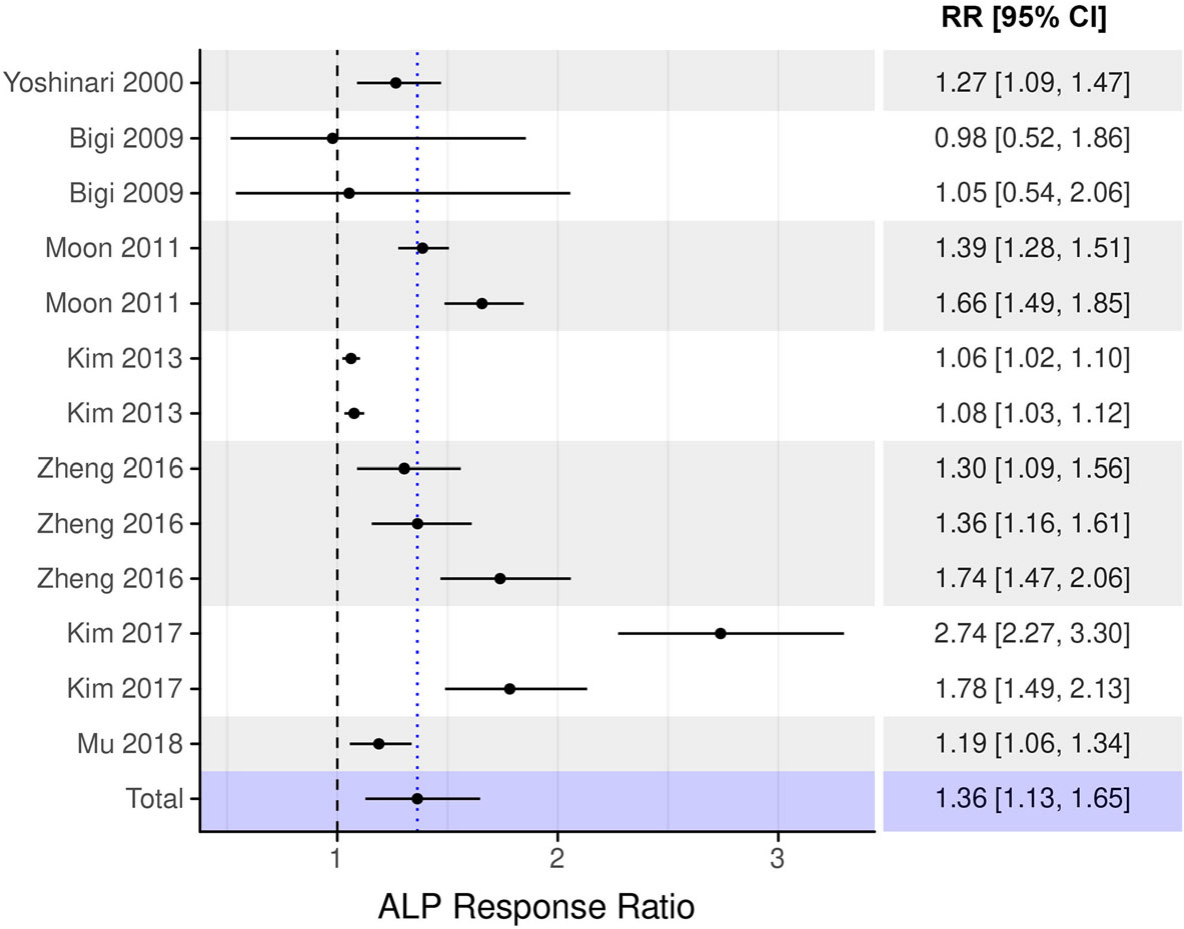
Forest plot of the association between bisphosphonate coating and ALP response ratio after 7 days. ALP response ratio was calculated as the ratio of ALP activity measured in the treatment group to that measured in the control group. Response ratios > 1 indicate higher ALP activity in the treatment group as compared to the control group. RR – response ratio; CI – confidence interval

**Fig. 3:**
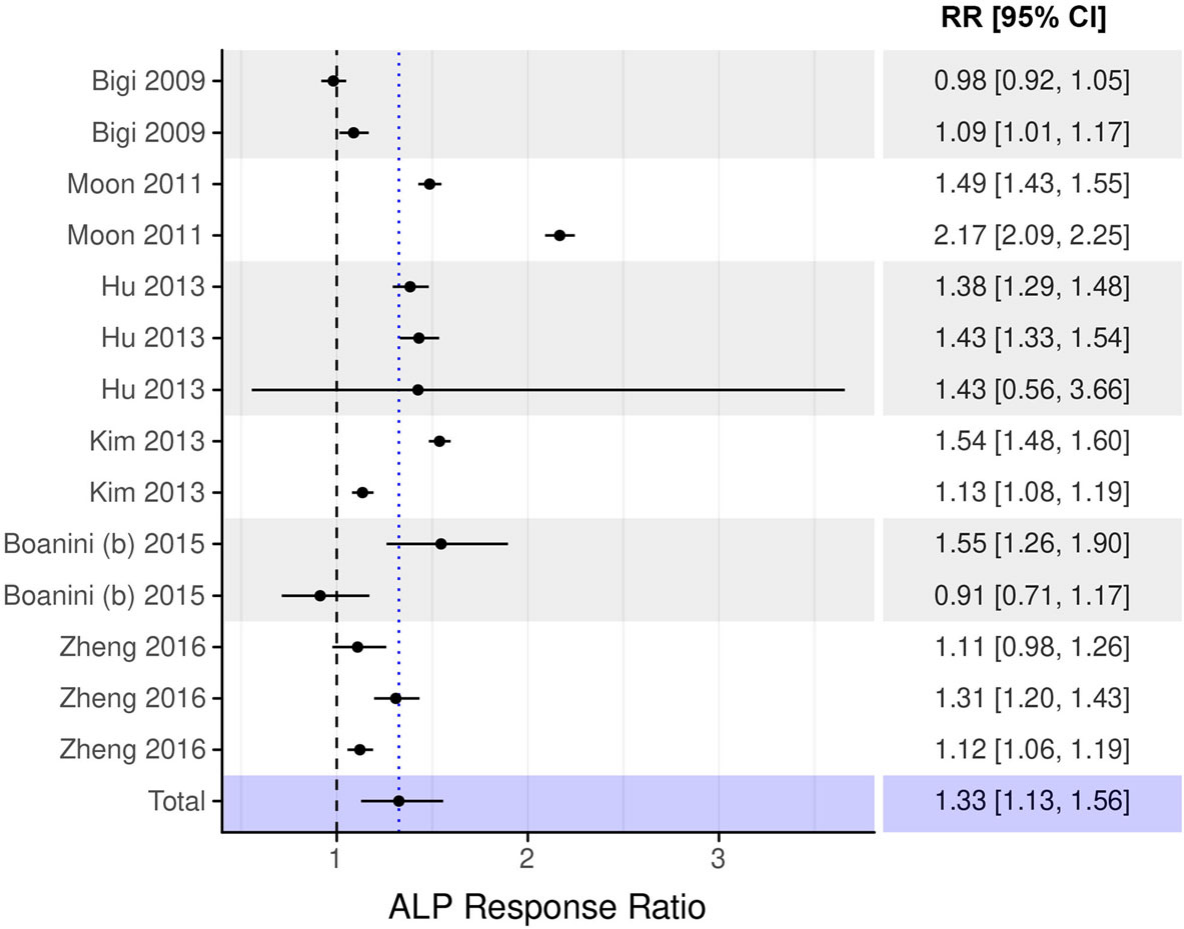
Forest plot of the association between bisphosphonate coating and ALP response ratio after 14 days. ALP response ratio was calculated as the ratio of ALP activity measured in the treatment group to that measured in the control group. Response ratios > 1 indicate higher ALP activity in the treatment group as compared to the control group. RR – response ratio; CI – confidence interval

**Fig. 4:**
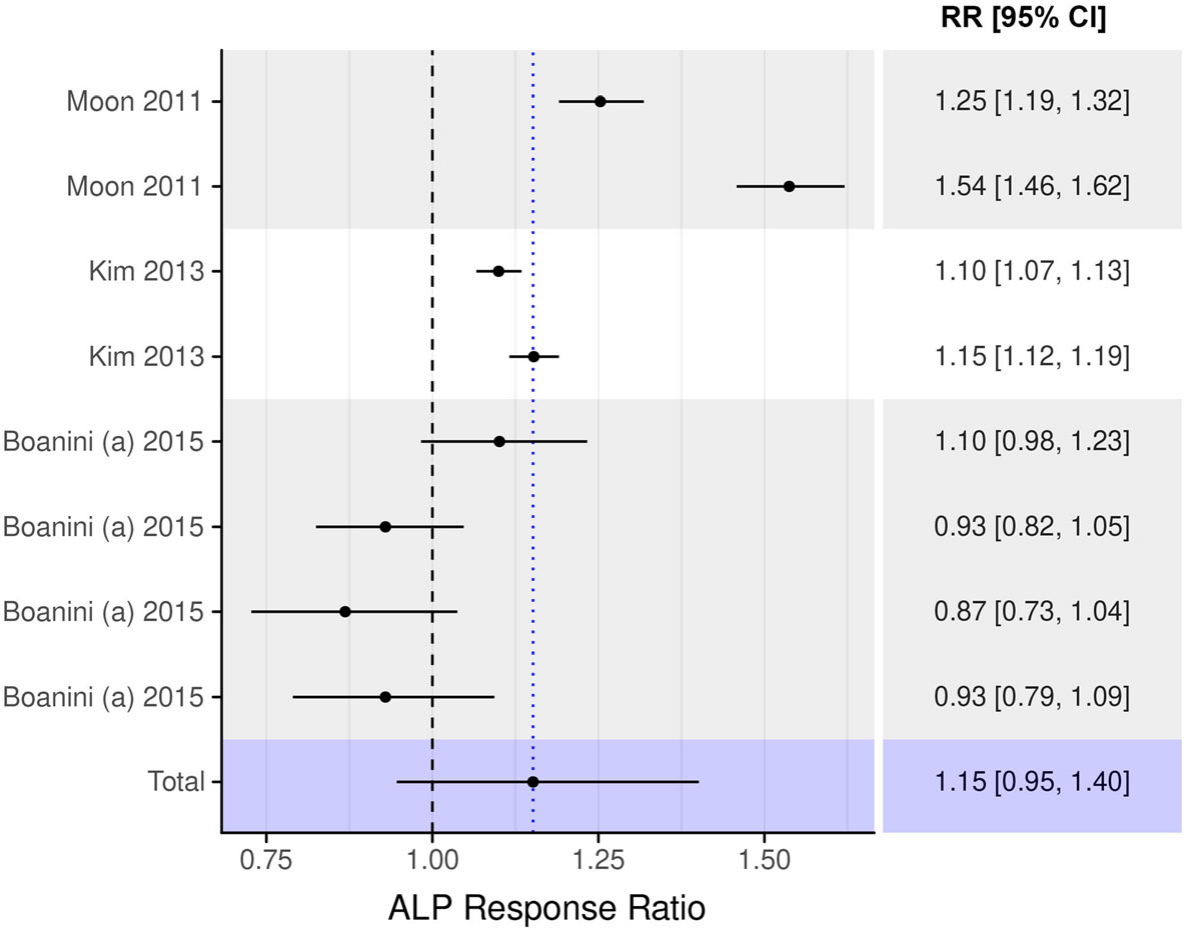
Forest plot of the association between bisphosphonate coating and ALP response ratio after 21 days. ALP response ratio was calculated as the ratio of ALP activity measured in the treatment group to that measured in the control group. Response ratios > 1 indicate higher ALP activity in the treatment group as compared to the control group. RR – response ratio; CI – confidence interval

The results of the meta-analysis at days 7, 14, and 21 days are presented in Figs. 2, 3, and 4. At day 7, meta-analysis revealed a 36.3% higher ALP activity in osteoblasts following cell culture on bisphosphonate-coated titanium surfaces compared to control (T vs C: beta coefficient = 1.363, 95%-CI from 1.128 to 1.648, *p*-value = 0.001; Fig. 2). Significant effect was still observed after 14 days of cell culture, exhibiting 32.5% higher ALP activity in bisphosphonate-coated groups vs. non-treated titanium surfaces (T vs. C: beta coefficient = 1.325, 95%-CI from 1.128 to 1.557, *p*-value < 0.001; Fig. 3). The 21-day model showed an about 15% higher ALP activity, but the effect was not statistically significant (T vs. C: beta coefficient = 1.152, 95%-CI from 0.946 to 1.401, *p*-value = 0.159; Fig. 4).

The forest plots performed in the subgroups depending on the bisphosphonate type, coating, and cells are presented as additional files.

### Bias assessment

**Fig. 5:**
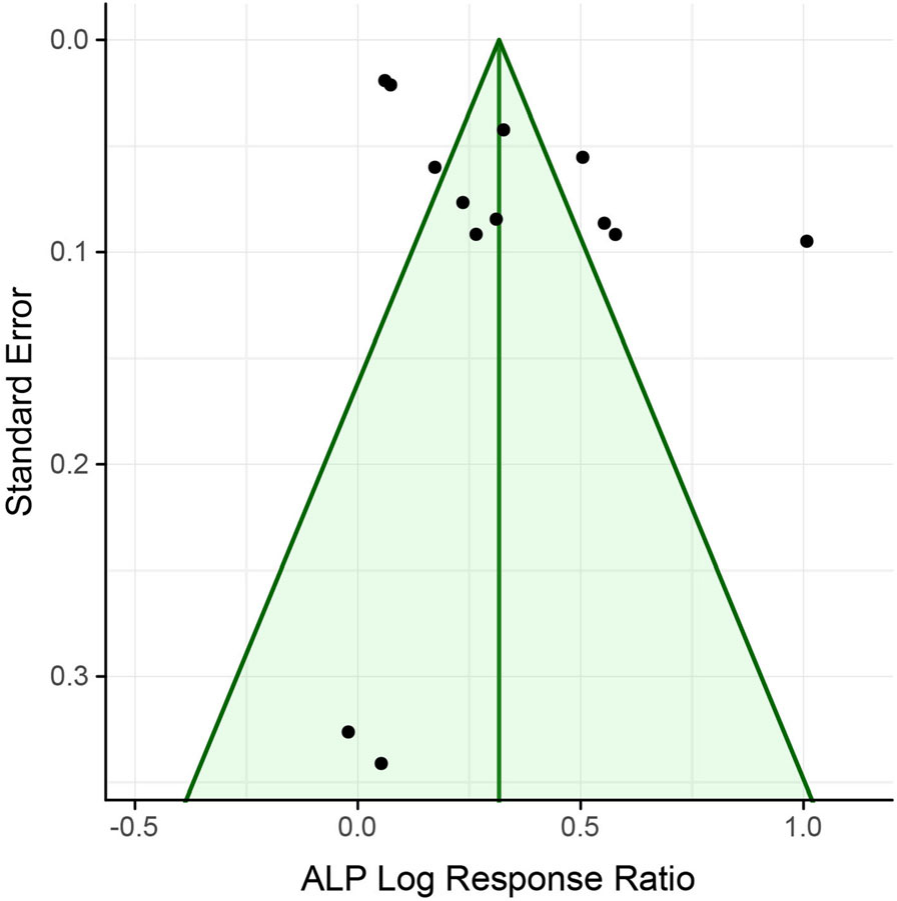
Funnel plot of the association between bisphosphonate coating and ALP response ratio after 7 days

Figure 5 shows the funnel plot referring to 7th day. This plot exhibited no asymmetry (Kendall’s tau is 0.23, p-value 0.306. p-value from Egger’s test is 0.412. I^2^ is 96.52). No further indication of relevant publication bias was found. The Funnel plots for days 14 and 21 exhibited a similar distribution (data not shown due to the low number of studies for these time points).

## Discussion

Biological effects of bisphosphonates are mainly related to inhibition of osteoclasts activity, whereas their impact on osteoblasts is less obvious. According to current literature, in vitro data on the effect of bisphosphonates on ALP activity in osteoblasts are inconsistent [27, 29]. Similarly, some discrepancy exists among the studies investigating osteoblasts growing on bisphosphonate coated titanium surfaces: many studies indicate a significant increase in ALP activity following osteoblast culture, but some reports show no significant effect [40, 45, 48]. Our meta-analysis showed that bisphosphonate coatings significantly improve ALP activity suggesting that the biological effects of bisphosphonates might also be partially contributed by promoting osteoblasts function. These findings are further supported by a pre-clinical study demonstrating an enhancement of peri-implant bone density and an increased mechanical fixation of bisphosphonate-coated dental implants in the rat model [51]. Moreover, our results are also in agreement with clinical studies that report an improvement of osseointegration parameters, better implant stability, and reduced peri-implant bone loss after local bisphosphonates application [52, 53]. According to the present findings, studies included for meta-analysis that investigated alendronate or pamidronate coating of titanium surfaces increased ALP activity in osteoblasts. Interestingly, one study utilizing zoledronate as coating showed lower ALP activity results compared to untreated control [41]. This however might be explained by the fact that zoledronic acid might exert toxic effects on osteoblasts at higher concentrations.

Osseointegration is a complex process involving a plethora of different cells and mechanisms (36) and can only be partially reflected in in vitro settings. Alkaline phosphatase is an early marker of osteoblast differentiation and bone formation [54]. Further indicators for osteoblast differentiation comprise osteocalcin (OC), type I collagen, or runt-related transcription factor 2 (Runx2) expression [25, 55]. However, the ALP activity was the most frequently investigated parameter in studies evaluating the osteogenic potential of titanium surface coatings with bisphosphonates. Among the studies included for meta-analysis, the expression of OC reflecting late osteogenic differentiation [56] was assessed in only four papers [40, 45, 47, 50], type I collagen expression was determined in three studies [40–42], whereas none of the studies performed evaluation of Runx2. Bisphosphonates coated implants demonstrated an increase in the expression of OC or type I collagen compared to control in all studies investigating these parameters and thus support our conclusion about a beneficial effect of bisphosphonate coating on osteogenic differentiation in vitro*.*

To assess publication bias, Egger’s test, as well as Kendall’s tau, were applied to evaluate funnel plot asymmetry. No funnel plot asymmetry was detected. To the best of the authors’ knowledge, there is no validated bias risk assessment tool available for in vitro studies. However, it has to be considered that also other factors, such as differences in study quality or study heterogeneity, could lead to asymmetry in funnel plots.

One possible heterogeneity source is the use of four different cell types by the included studies. Some studies used MG-63 human osteosarcoma cells as osteoblasts model [40, 41, 43, 46, 57]. These cells largely reflect many properties of primary osteoblasts [58]. Other studies used the murine calvarial pre-osteoblast cell line MC3T3-E1 [44, 47, 50]. Although these cells are widely used in material research, a recent study suggests that the performance of these cells might be different in the different subclones [59]. Two studies used primary cells isolated from rat calvaria [48, 49], and one study used mesenchymal stem cells derived osteoblasts [42]. Although the primary cells most adequately reflect the physiological situation, their performance might depend on the donor and the isolation method [60].

There is no standardized, validated tool for the risk of bias assessment for in vitro studies, and therefore we could not perform bias assessment by the traditional algorithm. Instead, we focused on the question if and how some crucial parameters were controlled in the included studies. In eight out of 11 papers, the water contact angle measurements have not been performed [40–43, 45, 46, 48, 49]. We considered this parameter for study quality assessment because it reflects the hydrophilicity of titanium surfaces, which enhances the alkaline phosphatase expression of osteoblasts [61, 62]. Three studies demonstrated a significant decrease in contact angle after bisphosphonate coating procedure [44, 48, 50], which might contribute to the improved osteoblasts differentiation.

Titanium surface microscale roughness is a further important parameter influencing osteoblast response and ALP activity [8, 63]. Coating procedures utilizing diamond-like carbon (DLC) may alter titanium surface properties and influence surface topography and roughness parameters [64]. Five out of 11 included studies investigated the effect of bisphosphonate coating. They found no significant changes in roughness parameters, including an arithmetic average of the *roughness* profile (Ra) and further parameters such as root mean square roughness (Rq) or maximum height of the profile (Rt) upon coating procedure [40–42, 48, 50].

In nine studies [40–42, 44, 46–50] titanium surface coating was done in combination with other components, such as hydroxyapatite (HA). Since HA is known to promote ALP activity in osteoblasts [65, 66], we did not consider pristine titanium but surfaces that were coated with the respective components as control. In terms of quality of ALP activity measurement, we regarded a normalisation of ALP data to cell number or protein amount as correct, instead of indicating absolute value. Such normalization of ALP activity measurement was performed in 8 studies [44–50].

It has to be also considered that coatings may exert biological effects only within a limited time period, as long as the drug or substance remains attached to the surface [67]. The assessment of the bisphosphonate coating stability in vitro was performed only by 6 out of 11 studies included in meta-analysis. The quantity of bisphosphonate released ranged from almost no measurable amounts [44] up to 40% of the initially immobilized substance [50]. The instability of coating could partially underlie the fact that its effect on the ALP activity was not significant after 21 days. Furthermore, also the bisphosphonate concentrations used for the coatings varied among the different studies, which could affect alkaline phosphatase activity to an unequal extent. It has to be taken into account that bisphosphonates at higher concentrations may also have cytotoxic effects on osteoblasts in vitro inhibit their viability [68, 69]. One study observed concentration-dependent inhibition of osteoblasts viability on bisphosphonate coated surfaces [41]. In contrast, other studies showed beneficial effects of bisphosphonates on osteoblast proliferation/viability [40, 46].

An increased risk of developing osteonecrosis of the jaw (ONJ) is an undesirable side effect of systemic bisphosphonates therapy. Invasive surgical procedures like tooth extraction or dental implant placement have been demonstrated to increase the risk of ONJ development. The prevalence of ONJ induced by systemic bisphosphonates application depends on the bisphosphonates type, dosage and treatments duration [70]. However, the risk of ONJ upon local application of bisphosphonates coated surface needs to be further assessed. Local delivery might require lower amount of bisphosphonates compared to the systemic therapy and therefore be associated with the lower risk of ONJ.

A possible limitation of our study is that the search for grey literature was not included, as we considered quality assessment achieved by the peer-review process indispensable. This process assesses the experimental protocol, which is essential especially for in vitro studies. As another study limitation, it has to be taken into account that restriction to literature in English might bias the outcome of the meta-analysis. However, publications in English have undergone an international peer-review process, thus possibly meeting higher quality standards than reviewing on the national level. A further limitation of the present study is that its review protocol was not published in any platform, which could be considered less transparent compared to studies with published protocols.

## Conclusion

In conclusion, our systematic review and meta-analysis showed that bisphosphonate coating of titanium surfaces exerts beneficial effects on osteogenic parameters in osteoblasts in vitro. Further studies are required to elucidate the underlying biological mechanisms that are initiated by bisphosphonate coatings of dental implants during the process of osseointegration and validate their clinical application in dental implantology.

## Supplementary information

**Additional file 1.** Forest plots depending on type of bisphosphonate, coating specification, and cells for ALP activity after 7 days.

**Additional file 2.** Forest plots depending on type of bisphosphonate, coating specification, and cells for ALP activity after 7 days.

**Additional file 3.** Forest plots depending on type of bisphosphonate, coating specification, and cells for ALP activity after 7 days.

## Data Availability

The authors declare that they are in possession of complete data on the basis of which calculations have been performed. The authors will make data available upon request. For this purpose, interested party is suggested to contact authors‘institution directly: Division of Conservative Dentistry and Periodontology, University Clinic of Dentistry, Medical University of Vienna, Sensengasse 2a, 1090 Vienna, Austria.

## Abbreviations

ALP: Alkaline phosphatase
BIC: Bone to implant contact
DLC: Diamond-like carbon
HA: Hydroxyapatite
MSCs: Mesenchymal stem cells
OC: Osteocalcin
ONJ: Osteonecrosis of the jaw
Ra: *Roughness* profile
Rq: Root mean square roughness
Rt: Maximum height of the profile
Runx2: Runt-related transcription factor 2
