# Supplementary figures and images for: Effect of bisphosphonate treatment of titanium surfaces on alkaline phosphatase activity in osteoblasts: a systematic review and meta-analysis
Source: BMC Oral Health. 2020 Apr 25;20:125. doi: 10.1186/s12903-020-01089-4 (PMC7183598; doi:10.1186/s12903-020-01089-4)

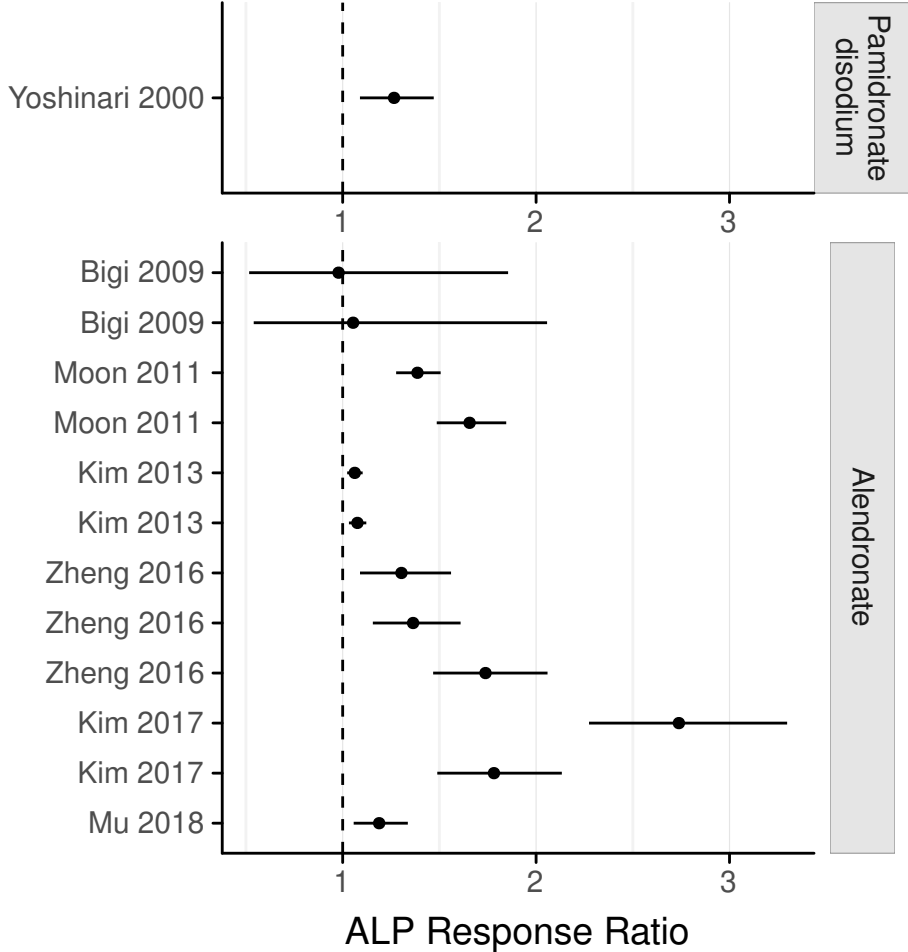

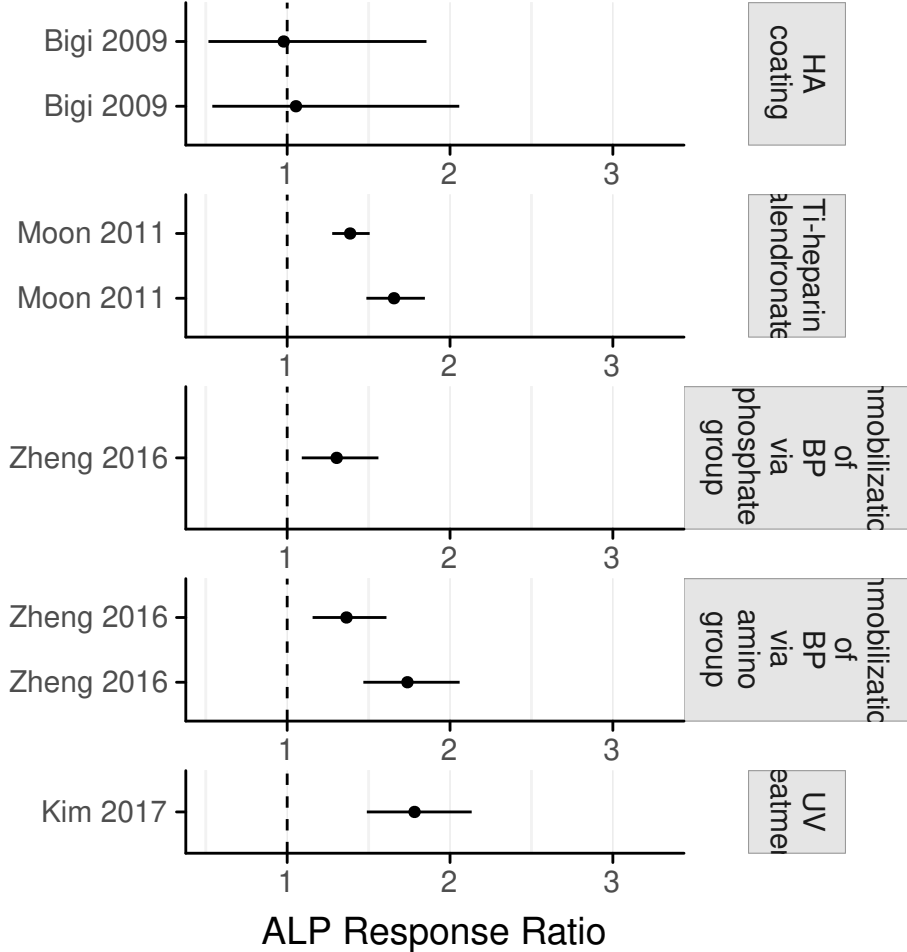

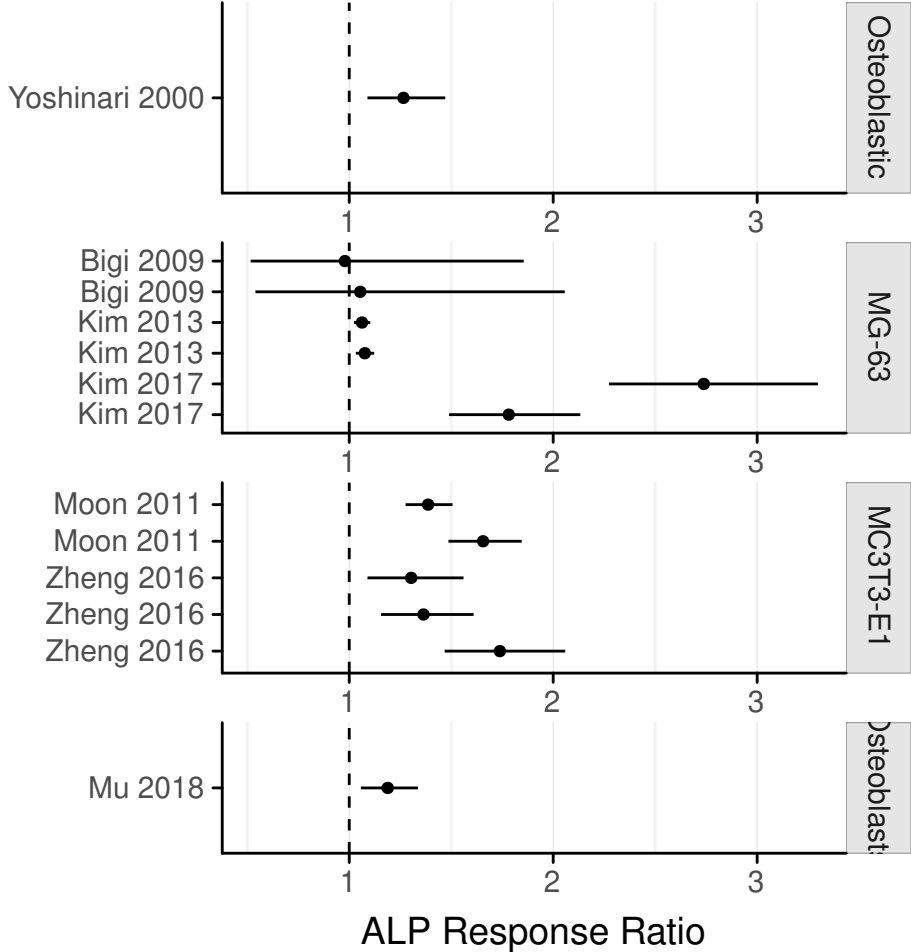

Supplement: Supplementary file 1 — Additional file 1. Forest plots depending on type of bisphosphonate, coating specification, and cells for ALP activity after 7 days. [file 12903_2020_1089_MOESM1_ESM.pdf]

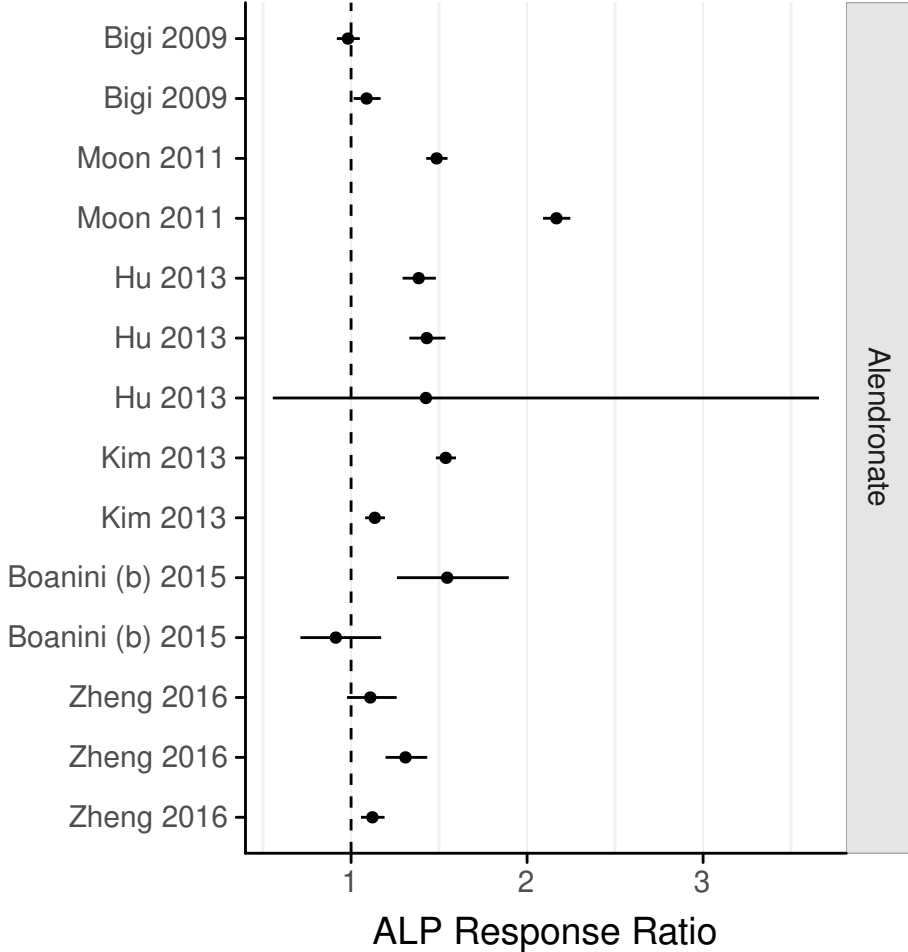

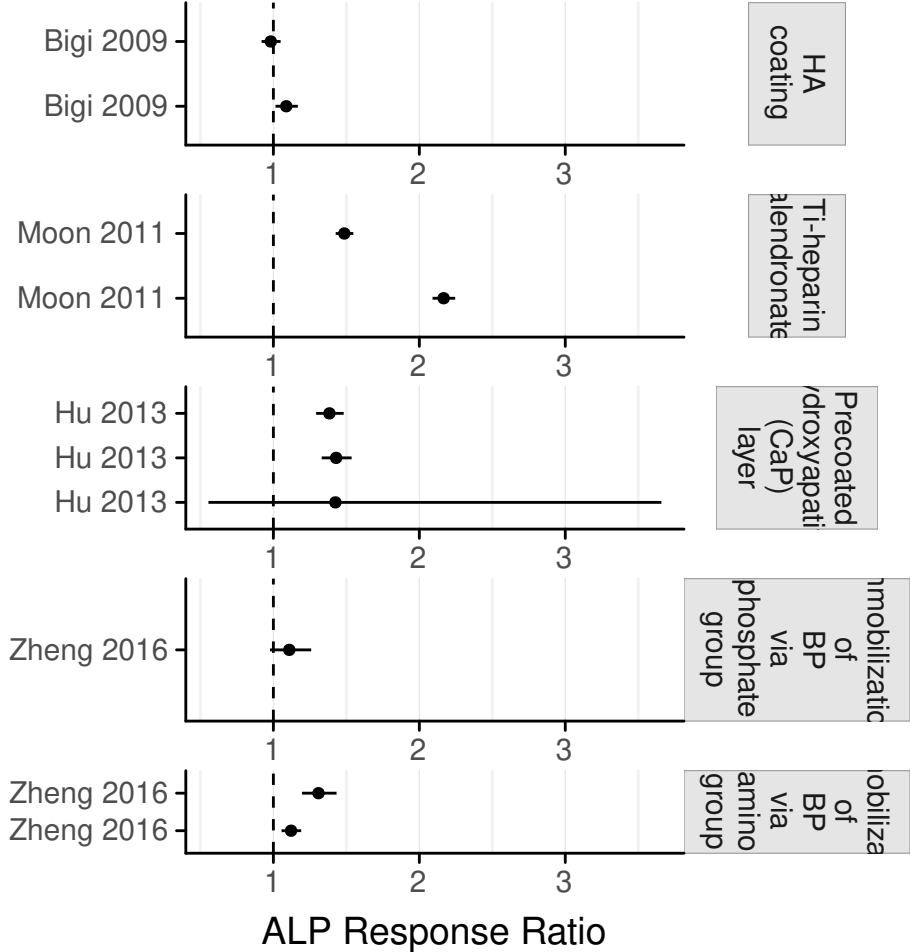

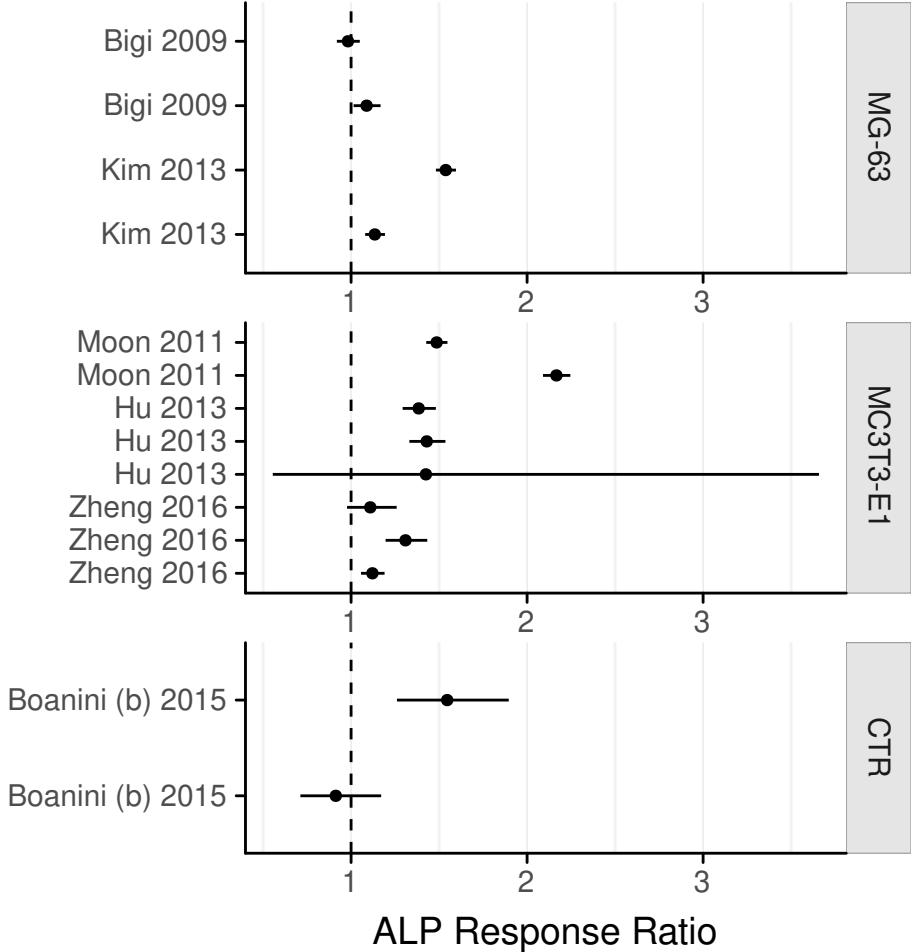

Supplement: Supplementary file 2 — Additional file 2. Forest plots depending on type of bisphosphonate, coating specification, and cells for ALP activity after 7 days. [file 12903_2020_1089_MOESM2_ESM.pdf]

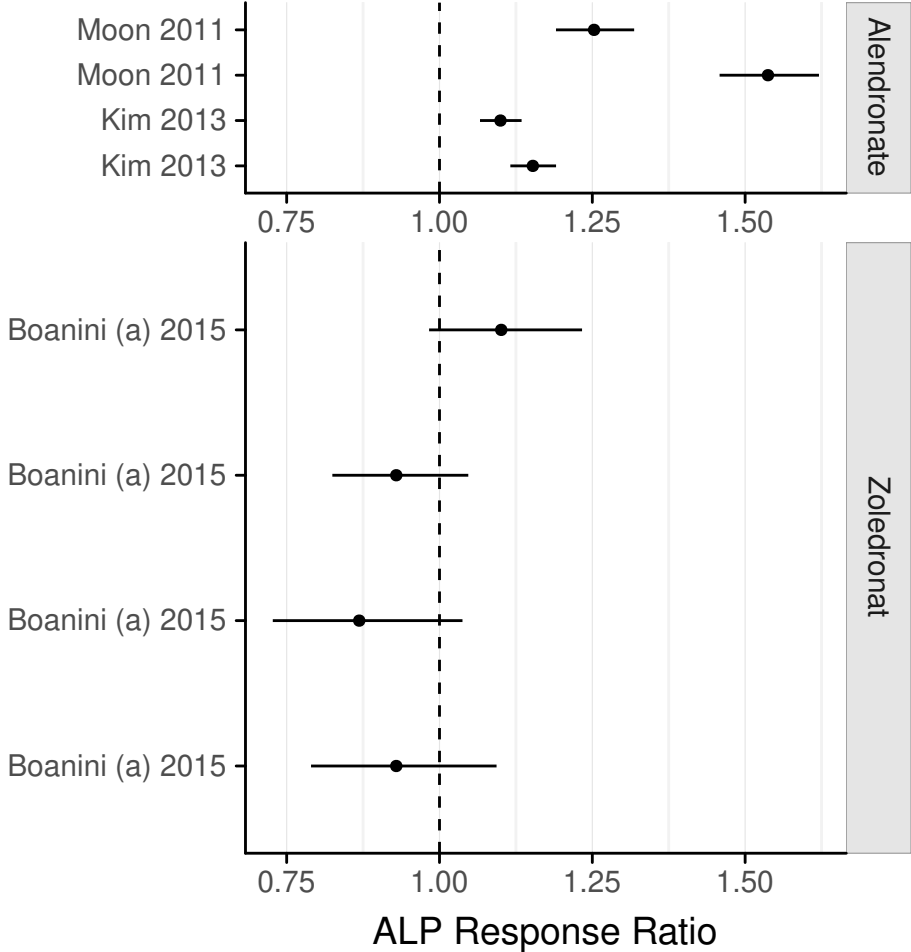

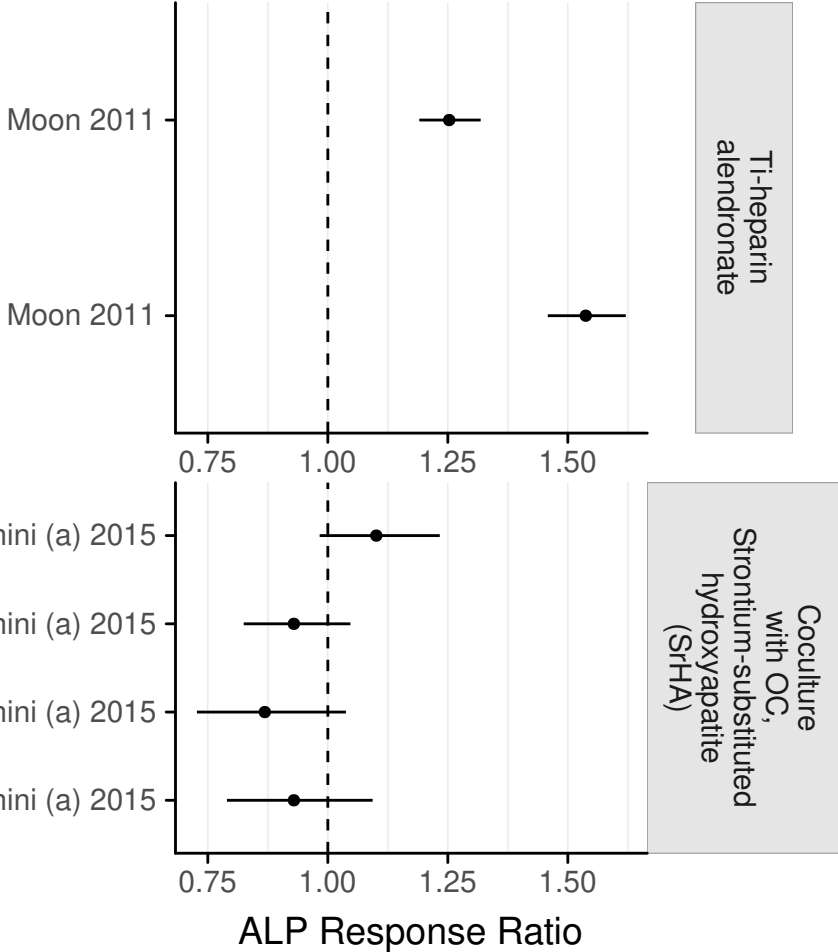

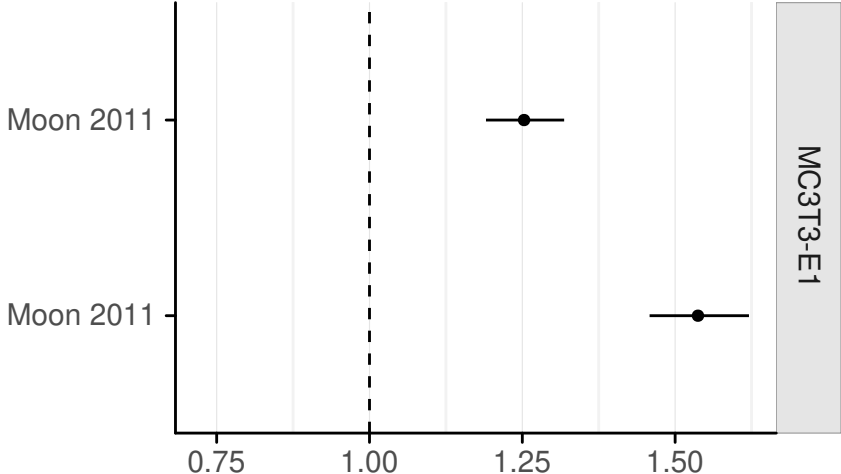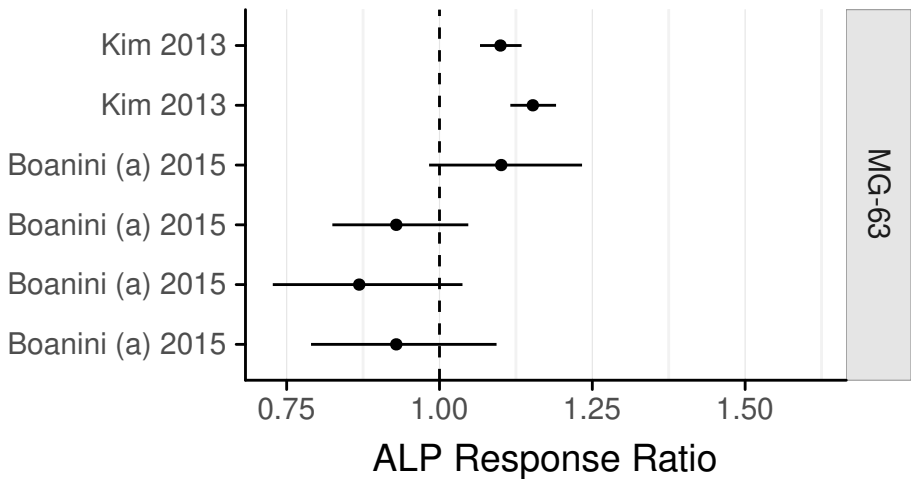

Supplement: Supplementary file 3 — Additional file 3. Forest plots depending on type of bisphosphonate, coating specification, and cells for ALP activity after 7 days. [file 12903_2020_1089_MOESM3_ESM.pdf]
